# Analysis of Reported Voting Behaviors of US Physicians, 2000-2020

**DOI:** 10.1001/jamanetworkopen.2021.42527

**Published:** 2022-01-10

**Authors:** Ahmed Ahmed, Fouad Chouairi, Xiaojuan Li

**Affiliations:** 1 Harvard Medical School, Boston, Massachusetts; 2 Department of Internal Medicine, Duke University Medical Center, Durham, North Carolina; 3 Department of Population Medicine, Harvard Medical School and Harvard Pilgrim Health Care Institute, Boston, Massachusetts

## Abstract

This cross-sectional study assesses the trends in physician-voter turnout and reported reasons for not voting in midterm and general US elections from 2000 to 2020.

## Introduction

Physicians’ engagement with the political process, particularly through voting, plays an important role in shaping public policy, affecting patient health and clinical practice.^1^ Historically, physicians in the US voted at lower rates than the general population in elections.^2^ However, physician voter engagement may have evolved, particularly given the changing political landscape and importance of US health care reform, as highlighted by the COVID-19 pandemic and differing policy proposals between the 2 major political parties. We assessed the trends in physician-voter turnout and reported reasons for not voting from 2000 through 2020.

## Methods

This cross-sectional study was exempted from review and informed consent by the institutional review board at Harvard Pilgrim Health Care because of the use of publicly available, deidentified data. This study followed the Strengthening the Reporting of Observational Studies in Epidemiology (STROBE) reporting guideline.

We used the US Census Bureau Current Population Survey Voting and Registration Supplement data.^3^ This survey is administered biennially to 60 000 households and collects information on voter participation and registration in the weeks following an election day. The response rates ranged from 79% to 92% in 2000 to 2020.

We estimated marginal probabilities of voter turnout (ie, the proportion of eligible individuals who voted) in physicians and the general population in each election, respectively, using multivariable logistic regression and adjusting for demographic characteristics strongly associated with voting.^4^ We evaluated differences in voter turnout between physicians and the general population using risk ratios (RRs) and χ^2^ tests, both pooled and individually in each election. We examined the impact of sex, age, and availability of no-excuse mail-in voting on physician voter turnout. To examine barriers to voting among physicians, we calculated the proportions of eligible physicians who cited each reason for not voting and not registering to vote, respectively. All estimates were weighted to be nationally representative.

All analyses were conducted in R version 3.6.3 (R Foundation). Tests were 2-sided, and a *P* value less than .05 was determined to be statistically significant. The study period was November 2000 to November 2020.

## Results

Survey respondents from 2000 to 2020 included 4330 physicians and 1 438 809 nonphysicians. Among physicians, 1449 (33.5%) were female, 211 (4.9%) were Black physicians, 223 (5.2%) were Hispanic physicians, 3354 (77.5%) were White physicians, and 3598 (83.1%) were aged between 30 to 64 years. Physicians and nonphysicians differed in most demographic characteristics.

Adjusted for demographic differences, pooled physician voter turnout was lower than the general population (57.4% vs 63.4%; RR, 0.91 [95% CI, 0.87-0.94]; *P* < .001). However, individual election results showed changes in this trend, especially in recent elections (Figure). Physicians had similar voter turnout as the general population in 2018 (RR, 1.00 [95% CI, 0.90-1.10]; *P* = .97) and higher turnout in 2020 (RR, 1.09 [95% CI, 1.00-1.18]; *P* = .03). Gaps in turnout between physicians and the general population were narrower in presidential elections than in midterm elections. Among physicians, voter turnout did not differ by sex but increased with age group with Generation X (born 1965 to 1979; RR, 1.25 [95% CI, 1.14-1.35]), Baby Boomer (born 1946 to 1964; 1.30 [95% CI, 1.17-1.43]), and Silent Generation (born 1925 to 1945; RR, 1.44 [95% CI, 1.09-1.80]) physicians more likely to vote than Millennial participants (born 1980 to 1994). Turnout was higher in states that offered no-excuse mail-in voting than those that did not (RR, 1.08 [95% CI, 1.03-1.13]; *P* = .003).

**Figure.**
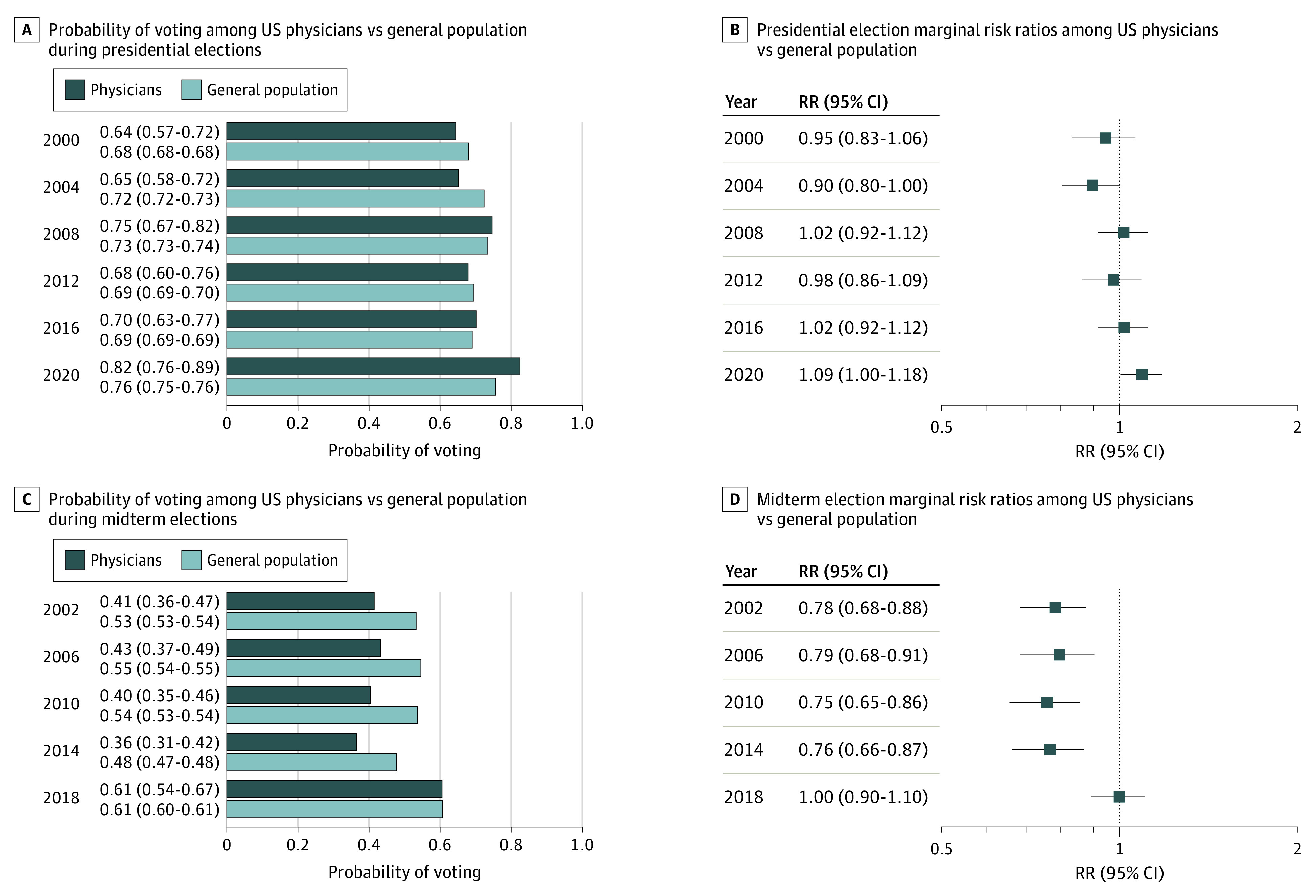
Marginal Estimated Probabilities and Risk Ratios of Voter Turnout Comparing US Physicians With the General Population, 2000-2020 The analyses are based on data from the Current Population Survey Voting and Registration Supplement. To be consistent with the US Census Bureau’s approach, we classified nonresponses and responses of no or do not know to the voter questions as did not vote. All estimates provided in both panels were survey-weighted and adjusted for differences in demographic characteristics. Using a regression-based equivalent approach of standardization, these estimated risk ratios quantify the mean association between the physician occupation and voting participation in the overall population.

Common barriers to voting cited by eligible physicians were not being registered to vote and being too busy or having a conflicting work schedule (Table). The most common reasons for not registering were not meeting the registration deadline and not being interested in politics.

**Table.** Common Reasons Why Eligible US Physicians Did Not Vote or Register in Presidential and Midterm Elections, 2000-2020

Year	Percentage of eligible physicians, estimate (95% CI)^a^									
	Reasons for not voting					Reasons for not registering				
	Not registered^b^	Too busy, conflicting work schedule	Away from home	Felt my vote would not make a difference	Other^c^	Did not meet registration deadline	Not interested in politics	Felt my vote would not make a difference	Did not live here long enough	Other^d^
**Presidential election**										
2000	6.2 (3.2-9.1)	2.3 (0.5-4.1)	2.0 (0.1-4.0)	0.6 (0.0-1.8)	1.0 (0.0-2.1)	NA^e^	NA^e^	NA^e^	NA^e^	NA^e^
2004	7.8 (4.6-10.9)	1.2 (0.1-2.3)	0	0.1 (0.0-0.2)	2.7 (0.8-4.7)	2.1 (0.2-3.9)	2.0 (0.5-3.5)	0.5 (0.0-1.3)	1.1 (0.0-2.5)	1.6 (0.2-3.0)
2008	2.9 (1.0-4.8)	1.2 (0.0-2.4)	0	0.3 (0.0-0.8)	1.8 (0.2-3.4)	0.7 (0.0-1.7)	0.3 (0.0-1.0)	0.6 (0.0-1.5)	0.4 (0.0-1.1)	0.9 (0.0-1.9)
2012	5.7 (3.2-8.3)	1.7 (0.1-3.2)	0.5 (0.0-1.6)	0.4 (0.0-1.1)	0.9 (0.0-1.9)	2.2 (0.5-3.8)	0.6 (0.0-1.4)	0	0.8 (0.0-1.8)	1.6 (0.2-3.0)
2016	5.2 (2.6-7.8)	1.0 (0.0-2.1)	0.7 (0.0-2.0)	0.2 (0.0-0.5)	1.6 (0.2-3.1)	1.8 (0.2-3.3)	1.9 (0.2-3.6)	0	0.3 (0.0-0.8)	0.9 (0.0-2.1)
2020	3.4 (1.2-5.6)	1.4 (0.0-2.8)	0	0.4 (0.0-1.1)	1.0 (0.0-2.1)	0.4 (0.0-1.3)	1.0 (0.0-2.2)	0	0.3 (0.0-0.9)	1.6 (0.0-3.2)
**Midterm election**										
2002	14.1 (10.3-17.9)	9.9 (6.8-13.0)	1.5 (0.3-2.6)	1.4 (0.0-2.7)	3.5 (1.5-5.5)	NA^e^	NA^e^	NA^e^	NA^e^	NA^e^
2006	9.3 (5.6-12.9)	7.7 (4.9-10.6)	1.1 (0.0-2.1)	1.9 (0.0-3.5)	3.6 (1.6-5.7)	2.8 (1.0-4.6)	3.1 (1.1-5.1)	0.1 (0.0-0.2)	0.4 (0.0-0.8)	1.3 (0.1-2.6)
2010	8.7 (5.5-12.0)	13.8 (9.6-18.0)	1.7 (0.3-3.0)	1.3 (0.0-2.7)	1.9 (0.6-3.3)	1.9 (0.2-3.6)	1.5 (0.1-2.8)	0.7 (0.0-1.6)	0.8 (0.0-1.9)	3.9 (1.7-6.0)
2014	8.3 (5.3-11.3)	9.2 (6.1-12.3)	2.6 (1.0-4.2)	1.6 (0.3-2.9)	4.7 (2.4-7.0)	2.3 (0.6-4.0)	2.6 (1.0-4.3)	0.3 (0.0-0.9)	0.6 (0.0-1.5)	2.1 (0.6-3.6)
2018	4.7 (2.3-7.0)	3.7 (1.8-5.6)	1.5 (0.2-2.9)	0.1 (0.1-0.3)	1.9 (0.5-3.3)	0.8 (0.0-1.8)	1.2 (0.0-2.2)	0.0 (0.0-0.1)	0.3 (0.0-0.9)	2.0 (0.5-3.6)

Abbreviation: NA, not available.

^a^ Denominator for percentages reported is the number of physicians who were eligible to vote in each election year; the proportions are survey weighted to be nationally representative.

^b^ To be consistent with the US Census Bureau’s approach, we classified nonresponses and responses of either no or do not know to the registration questions as not registered.

^c^ Other reasons for not voting include: illness or disability, forgot to vote, transportation problems, didn’t like candidates, registration problems (didn’t receive absentee ballot, not registered in current location), bad weather conditions, inconvenient polling place, concerns about COVID-19 pandemic, and other reasons. They were combined into a single category because of the small number of physicians who cited them.

^d^ The response other was an answer option in the survey; the survey did not capture what other reasons respondents were referring to.

^e^ The survey question regarding why unregistered citizens did not register to vote was not included in the Current Population Survey in election years 2000 and 2002.

## Discussion

Voter turnout among US physicians has grown during the past 2 decades. Physicians were just as likely to vote as the general population in the 2018 midterm and more likely in the 2020 presidential election. These changes were likely multifactorial and may be related to health care reform and increased focus on physicians’ civic roles.^5,6^ Physicians in states that allowed no-excuse mail-in voting had a higher turnout. However, physicians still face barriers to voting, including registration and work schedule conflicts. Study limitations include potential reporting bias, overestimation of voter turnout by survey-based estimates, and limited representation of political participation with voting in nonprimary elections.
